# The Impact of Digital Screen Time on Dietary Habits and Physical Activity in Children and Adolescents

**DOI:** 10.3390/nu14142985

**Published:** 2022-07-21

**Authors:** Agata Rocka, Faustyna Jasielska, Dominika Madras, Paulina Krawiec, Elżbieta Pac-Kożuchowska

**Affiliations:** 1 Students’ Scientific Group, Department of Pediatrics and Gastroenterology, Medical University of Lublin, Racławickie 1, 20-059 Lublin, Poland; agatarocka2@gmail.com (A.R.); faustyna.piedel@gmail.com (F.J.); dkmadras@gmail.com (D.M.); 2 Department of Pediatrics and Gastroenterology, Medical University of Lublin, Racławickie 1, 20-059 Lublin, Poland; elzbietapackozuchowska@umlub.pl

**Keywords:** lifestyle, electronic devices, diet, obesity

## Abstract

Background: Over the last few decades, the time children spend using electronic devices has increased significantly. The aim of the study was to evaluate the impact of screen time on dietary behaviors and physical activity in children and adolescents. Methods: An online survey was conducted among parents of preschool and school-aged children during the COVID-19 lockdown in Poland. There were 3127 surveys used in the analysis. Results: Survey responses referred to 1662 (53%) boys and 1465 (47%) girls, with a mean age of 12.1 ± 3.4 years. During a routine weekday, most children (71%) spent >4 h on educational activities using electronic devices, and 43% of children spent 1–2 h using devices for recreational purposes. The majority of children (89%) were exposed to screens during meals, and ate snacks between main meals (77%). There was an association between screen time and the exposure to screens during meals, and between screen time and time spent performing physical activity. Conclusions: This study revealed that the majority of children were exposed to screens during meals, which is a risk factor of obesity. The promotion of the judicious use of digital devices and healthy dietary habits associated with the use of screens may be an important component of obesity prevention strategies.

## 1. Introduction

Although high-quality screen activities meet some education and entertainment needs, exposure to digital screens may be detrimental for children’s physical health, cognitive skills, and psychosocial development [1].

Therefore, screen time in childhood and adolescence should be managed, weighing risks versus benefits. The American Academy of Pediatrics (AAP) recommends that children younger than 18–24 months of age should not ever use screen media, while older children should not use these media for more than a 1 h daily [2]. According to the World Health Organization (WHO), infants within the first year of life should not be exposed to digital screens. Screen time for children aged from 2 to 5 years old should not exceed 1 h per day [3]. Similar recommendations were released by the Canadian Pediatric Society, stating that children younger than 2 years old should not use screens, and children aged 2 to 5 years old should have limited screen time to less than 1 h per day [1]. The guidelines released by the Australian Department of Health for children older than 5 years old recommend that recreational screen time should not exceed 2 h per day [4]. The German recommendations advise reducing screen time to a minimum. Infants and toddlers should not use electronic devices; preschool children should have limited screen time to 30 min daily; primary school children should be limited to 60 min daily; and adolescents should be limited to 120 min daily [5]. On the other hand, the Royal College of Pediatrics and Child Health (RCPCH) indicates that due to the lack of solid scientific evidence, it is not possible to formulate a universal cut-off for children’s screen time. Thus, the RCPCH recommends that families should negotiate screen time while considering individual child’s needs, the quality of digital activities, and the extent to which using electronic devices displace physical and social activities and sleep [6]. 

However, a recent systematic review which performed a meta-analysis of 63 studies revealed that only 24.7% of children younger than 2 years of age and 35.6% of children aged between 2 and 5 years old met screen time guidelines [7]. Differences were noticed in children’s adherence to appropriate screen time depending on the country [8].

It appears that the reasonable management of children’s screen time is now more challenging than ever. Due to the coronavirus disease 2019 (COVID-19) outbreak, social distancing restrictions have been introduced, which obliged children and adolescents to engage with remote learning, limited opportunities for physical activity, and exacerbated unhealthy eating habits [9,10,11]. Thus, it is particularly important to collect contemporary data regarding screen time and to find effective strategies to promote the judicious use of digital devices and implement healthy lifestyle habits from early childhood. The aim of this study was to evaluate the relationships between digital screen time and eating habits and physical activity in preschool and school-aged children during the COVID-19 lockdown in Poland.

## 2. Materials and Methods

A cross-sectional study using an anonymous online survey was conducted among parents of Polish preschool and school-aged children from 11 March 2021 to 20 March 2021. We requested all Polish Educational Offices to send the letter of invitation to participate in our study to kindergartens and schools, which spread this invitation among parents of pupils. Some of the Polish Educational Offices promoted information about this study on their websites. The invitation to the study included a link to the online questionnaire. 

A self-administrated caregiver-reported questionnaire was set up via Google Forms. The survey consisted of 34 closed and 11 open-ended questions on children’s demographic characteristics, screen time, dietary habits, physical activity and sleep in the recent three months. The authors’ questionnaire was available in Polish.

The final analysis included complete responses given by parents of children up to 18 years old who provided informed online consent prior to the survey. The exclusion criteria were as follows: responses given by parents of children older than 18 years old, incomplete data in the questionnaire, and lack of informed consent.

A total of 4437 responses were received, out of which 3127 met the inclusion criteria for the final analysis. There were 1310 polls excluded from the study due to a lack of informed consent for the study (n = 975), responses given by parents of children older than 18 years old (n = 121), and incomplete data (n = 214).

The statistical analysis was carried out using the Statistica version 13 program (StatSoft, Kraków, Poland). For nominal variables, to test differences between groups or relationships between parameters, the chi-squared test of homogeneity or independence was used. The statistically significant results were *p* < 0.05.

This study obtained approval of the Bioethics Committee of the Medical University of Lublin (KE-0254/15/2021). All participants provided online informed consent prior to survey initiation.

## 3. Results

Survey responses referred to 1662 (53%) boys and 1465 (47%) girls with age ranging from 1 to 18 years old. The mean age of the children was 12.10 ± 3.40 years old; the median was 12 years old. The majority of children lived in rural areas (n = 1603; 51.26%) and did not suffer from any underlying medical condition (n = 2489; 79.59%). The most common medical conditions in the study group were refractive errors (n = 245; 7.83%) and asthma (n = 90; 2.88%). General characteristics of the study group are summarized in Table 1.

The vast majority of parents declared that children used their own electronic devices (n = 2690; 86.00%). About one-third of respondents declared that they did not restrict access to electronic devices for their children (n = 1019; 32.00%).

**Figure 1:**
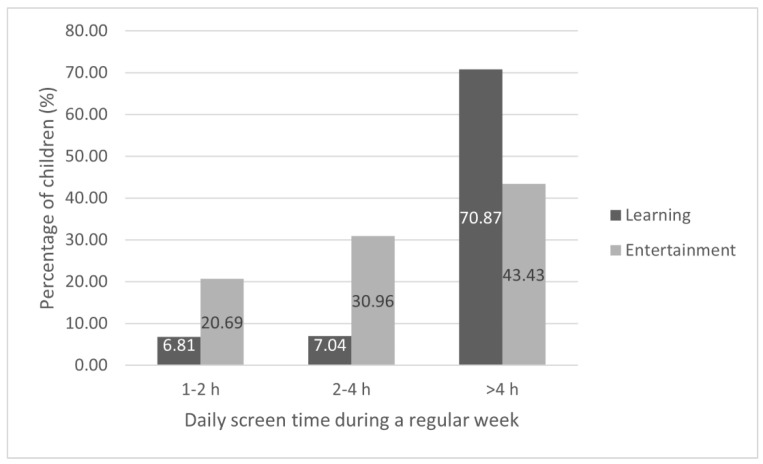
Screen time exposure during a regular weekday. Answers of parents who denied that their children were using digital devices for educational (15.20%) or recreational (4.92%) purposes are not presented.

**Figure 2:**
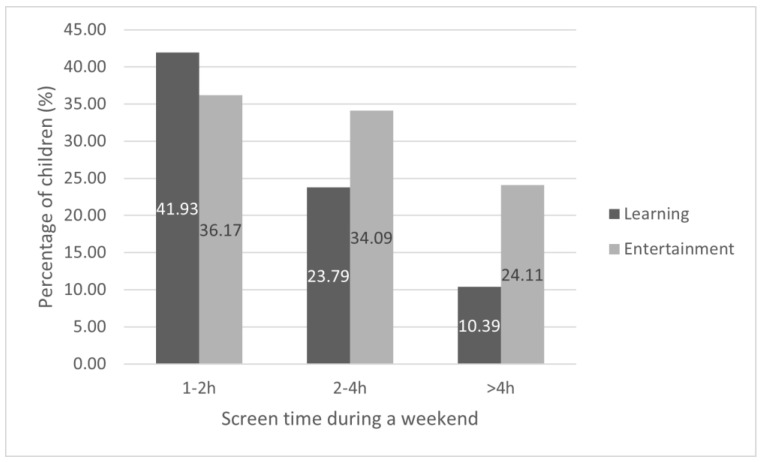
Screen time exposure during a weekend day. Answers of parents who denied that their children were using digital devices for educational (23.89%) or recreational (5.56%) purposes are not presented.

During a regular weekday, the majority of children (n = 2220; 71%) spent more than 4 h on educational activities using electronics. During the weekend, 10.39% (325) of children spent more than 4 h on educational activities using electronic devices. Figure 1 and Figure 2 show the time spent in front of screens on educational activities and entertainment during a regular weekday and weekend day. We also found an association between age and screen time, presented in Table 2, i.e., the older the child was, the longer screen exposure was reported. Answers of parents who denied that their children were using digital devices for educational or recreational purposes were not analyzed in these particular areas. There were 478 (15.29%) parents who declared that their children did not spend any time on educational activity using electronic devices during regular weekdays, and 747 (23.89%) during weekend days. There were 153 (4.92%) parents who declared that their children did not use electronic devices for recreational activities during regular weekdays, and 176 (5.63%) during weekend days.

Parents declared that children ate from 1 to 8 main meals per day (mean 4.43), with median 4.5. Moreover, 1788 (57%) of respondents declared that their children were eating meals at consistent times. The vast majority of children were exposed to digital screens during meals (n = 2782; 88.74%). Detailed analysis revealed that 345 (11.03%) children ate meals while using electronic devices every day, 775 (24.78%) did several times during a week, 1038 (33.20%) did several times during a month, and 617 (19.73%) rarely did. Another finding was an association between screen time and the exposure to screens during eating meals, as presented in Table 3. The longer screen time for educational and recreational activities was associated with more frequent exposure to screens during meals.

There were 2410 (77%) children who reported eating snacks between main meals. The most common types of snacks were fruits (n = 2170; 68.40%), cookies (n = 1247; 39.88%), and crisps (n = 1100; 35.18%). Notably, one respondent might have given more than one answer. The most common types of snacks eaten while using electronic devices are summarized in Table 4.

Daily beverage consumption during screen time was reported in 309 (9.88%) children. In 919 (29.40%) cases, parents reported that children consumed drinks while using devices several times per week, and in 1505 (48.13%) cases, several times per month. The majority of children and adolescents drank beverages while using electronic devices (n = 2733; 87.41%). The most common types of drinks chosen when using electronic devices were water (n = 2480; 79.31%), fruit juices (n = 1499; 47.94%), and tea (n = 1666; 53.28%). Types of drinks consumed while using electronic devices are presented in Table 5. One respondent may have given more than one answer.

Parents reported that the mean time their children spent performing daily physical activity during a regular weekday was 2.08 ± 1.8 h, with a median of 1.5 h. During a weekend day, children spent 2.19 ± 1.7 h performing physical activity, with a median of 2 h.

There was a statistically significant relationship between screen time and the time spent on physical activity (Table 6). Although the majority of children spend between 1 and 4 h daily on physical activity, children with longer screen time tend to be less physically active. Even though the survey was taken during the COVID-19 pandemic, 55.5% of respondents declared that their child was attending physical education classes. However, one needs to remember that during lockdown, physical education classes in some schools were conducted via online platforms. 

Interestingly, 2728 (87.00%) of parents believed there is an age limit until which child should not use electronic devices. Parents reported that the average age for adolescents making independent decisions about spending time in front of screens was 15 years.

## 4. Discussion

The dynamic developments in technology over last decades resulted in a significant increase in time spent using electronic devices by children [12]. Moreover, the COVID-19 pandemic exacerbated that trend [13]. A recent systematic review which performed a meta-analysis revealed that during the COVID-19 pandemic, there was a significant elevation of total screen time in 67% of children, and of recreational screen time in 60% of children [13]. The total screen time increased by 0.5 h per day (95% CI 0.3–0.9) in children younger than 5 years old, 0.9 h per day (95% CI 0.3–1.5) in adolescents aged 11 to 17 years old, and 1.4 h per day (95% CI 1.1–1.7) in children aged 6 to 10 years old was reported [13]. There was also an elevation of leisure screen time of 0.48 h daily (95%CI 0.29–0.67) in adolescents, 0.61 h daily (95% CI 0.4–0.82) in young children, and 1.04 h daily (95% CI 0.77–1.3) in primary aged children [13]. Moreover, there is also evidence for the decline in physical activity in children during the global pandemic [14]. Thus, it appears that due to the lockdowns, school closures and remote learning during the pandemic aggravated sedentary behaviors even in the youngest children, while physical activity has decreased [13,14,15,16].

To the best of our knowledge, this is one of the first studies among parents of children in all age groups which has evaluated the relationship between children’s digital screen time and lifestyle behaviors during the COVID-19 pandemic in Poland. In the present study, the vast majority of children used their own electronic device. During a regular weekday, 70% children spent more than 4 h daily on educational activities using electronics, and more than 40% spent more than 4 h daily on entertainment using electronic devices. During weekends, electronic devices were used for more than 4 h daily on educational activities by 10% of children, and on entertainment by almost 25% of children. In the study group, unhealthy eating habits were revealed, i.e., the vast majority of children were exposed to digital screens during meals and ate snacks between main meals. There was an association between screen time and the exposure to screens during eating meals. The median time spent performing physical activity was 1.5 h during weekdays and 2 h during weekend days. There was also a significant relationship between daily screen time and physical activity. 

In the current study, parents of preschool and school-aged children were included. Not only did the majority of children spend more than 4 h daily using electronic devices for educational activities, but a large proportion of them spent more than 2 h on entertainment-based screen time per day. Results from this study are consistent with previous findings. In a group of 166 children, Ferreira et al. showed that approximately 85% of children under 2 years of age and 80% of infants were exposed to electronic devices. Moreover, 79% of them spent up to 1 h per day in front of screens [17]. Kaur et al. also found that approximately 60% of children aged 2–5 years old had excessive screen time and did not follow AAP recommendations [18]. Thus, it now appears almost implausible to comply with recommendations on screen time for children [7,10,19,20].

Recent meta-analyses confirmed that a greater quantity of screen time is associated with an increased risk of obesity [21,22,23]. This phenomenon may be explained by the displacement of time for physical activity by screen-based sedentary behaviors [21,22,24]. Moreover, poor dietary habits, such as a higher intake of energy, more frequent consumption of fast food and sweets, and lower intake of fiber, vegetables, fruit, and fish were reported more often in children overexposed to digital devices [25,26]. One of the biggest contemporary health concerns is overweight and obesity in the pediatric population, which result from a lack of physical activity and unhealthy eating habits [27]. Notably, recent research has shown that the risk of obesity is higher in children who spend more time engaging in screen-based sedentary behaviors than in non-screen-based sedentary activities [28]. Sedentary digital media use in preadolescence was associated with an increased risk of overweight three years later [29]. Alturki et al. posited that having a smartphone was statistically significantly higher in a group of obese children than in children with normal weight [30]. Moreover, it has been revealed that during the COVID-19 pandemic, there was a trend in weight gaining, resulting in overweight and obesity in children and adolescents, which mostly affected those who were aged between 2 and 6 years [31]. 

Moreover, excessive exposure to digital screens negatively affects the duration and quality of sleep in childhood [21,22,32]. The link between screen time and poorer psychomotor and cognitive development has been also revealed [21,33]. On the other hand, although excessive screen time was associated with poorer language skills, the high-quality use of media was beneficial for child language [34]. There are some data also suggesting an association between screen time and the mental health of children and adolescents [21,22].

According to WHO guidelines, the median time spent performing physical activity in children aged 5 to 17 years should be 60 min per day [35]. In the present study, the median time for physical activity met WHO guidelines. Possible explanations of this finding may be the fact that the majority of children attended physical education classes. However, one needs to remember that during the lockdown, some of these school classes were conducted via online platforms, which raises concerns about the quality of children’s physical activity in these times.

A significant association was found between reduced physical activity and a long time spent watching TV, as well as a long time spent in front of any screen [10,36]. Decreased physical activity in school is associated with longer screen time. Moreover, lower physical activity can be associated with spending more time in front of a screen at night [37]. Recent studies have indicated that there is a correlation between screen addiction behaviors and decreased physical activity in young adolescents [10]. It is important to replace screen-based sedentary behaviors with physical activity [3]. There are data which show decreases in physical activity in children during the COVID-19 pandemic [16,29,38]. Comparable findings were observed in Polish children in studies by Brzęk et al. and Łuszczki et al. [11,39]. It should be highlighted that only 30.77% of children met the WHO physical activity guidelines before the COVID-19 pandemic [39].

In the current study, there was an association between screen time and physical activity. Similar results were obtained in other studies. Mineshita et al. found that children with a longer usage of electronic devices were more likely to spend less time on physical activities [40]. The use of television screens for more than 2 h may be related with a reduction in physical fitness among juveniles as well [41]. On the other hand, however, Dahlgren et al. did not find any correlation between screen time and physical activity in a group of adolescents [42].

It is recommended that children should not use a digital screen during meals [2]. The results of the current study revealed that most of the children were exposed to screens during meals. This is a significant factor which may trigger obesity and eating disorders. Using digital technologies that divert attention away from meal negatively affects eating behavior and results in an excessive energy intake [43]. Moreover, children using digital devices during meals tend to consume more junk food [44]. Yong et al. found that during a week-long study, 85.3% of participants used a smartphone at least once during a meal, with an average frequency of one in three meals where phones were used [45].

In the current study, there was an association between screen time and the frequency of exposure to screens when eating meals. Kristo et al. also found a significant correlation between children’s eating habits and the duration of tablet or smartphones use, but not with computer use [46]. Result of the study by Robinson et al. confirmed the influence of electronic devices on eating habits. It has been found that the more media devices children use during mealtime, the poorer the quality of the food [47]. Falbe et al. noted that longer screen time was associated with a lower consumption of vegetables and fruit, and a greater consumption of sweets, fast food, sweetened drinks, and salty snacks [48]. The results of the ALADINO study revealed that excessive screen time is correlated with a higher frequency of eating energy-dense and micronutrient-poor food, and a lower intake of fruit and vegetables [49]. According to Watts et al., time spent in front of a screen by adolescents was conducive to less healthy food choices [50]. Kelishadi et al. found that children who spent time in front of a screen for more than 4 h per day were more vulnerable to eating snacks compared with people with a daily screen time of less than 4 h [51]. Interestingly, in the present study, the majority of children ate snacks between main meals, most commonly fruit. 

The vast majority of children from the study group drank beverages while using digital devices, mostly water, tea, and juices. Börnhorst et al. showed that one additional hour of screen time increased the consumption of sugary soft drinks, diet soft drinks, and flavored milk [52]. The cross-sectional Spanish study revealed that screen time exposure for longer than 1 h can increase the frequency of sweets, fast food, and soft drink intake [53]. 

Most parents declared that their child used their own electronic device. Moreover, 87% of parents believed there was an age limit until which child should not use electronic devices. Parents reported that the average age at which children should make independent decisions about spending time in front of screens is 15 years old. British recommendations also highlight the need to set appropriate amounts of time for the independent use of electrical devices by children. It is important that the limits are set by parents and children together, and should be based on the child’s age. When limits are not respected, parents should clearly define the consequences. Moreover, the content of screen time also matters [54]. The question therefore arises as to what the best way is to educate parents on risks associated with prolonged screen time to enable them making rational decisions in this matter.

Our study has some limitations, including the limited response rate. This may be explained by the fact that the invitation to our study was sent indirectly to parents by Educational Offices. There was also a significant proportion of partial surveys which could not be used in the final analysis. Another limitation may be the fact that the study presents self-reported data by parents of children. Moreover, caution should be exercised when attempting to generalize the findings of the current study because it relates to an extreme event: the global COVID-19 pandemic. However, despite these limitations, this study identified important behaviors associated with screen time among children, which should be corrected through evidence-based interventions and prevention strategies.

## 5. Conclusions

In conclusion, screen time affects the lifestyle behaviors of children and adolescents. This study revealed that the vast majority of children were exposed to screens during meals. This behavior may increase risk of obesity due to higher energy intakes and the more frequent consumption of junk food. Starting in early childhood to promote the judicious use of digital devices and to build healthy dietary habits associated with the use of screens may be an important component in obesity prevention strategies: not only during the COVID-19 pandemic, but also in the future. Digital screen time may negatively affect physical activity. There is a need to develop effective strategies to limit excessive screen time and to promote healthy eating habits and physical activity in children. Understanding the mechanisms behind the impact of screens on children’s physical activity and eating habits may help to create targeted parental control plans, which could result in long-term effects such as reducing the risk of obesity in the future.

## Figures and Tables

**Table 1:** General characteristics of the study group.

Subject	Number of Subjects (n)	Percentage of Subject (%)
Sex		
Girls	1465	47.00
Boys	1662	53.00
Age groups [years]		
<3	20	0.64
3–6	146	4.67
7–11	1156	36.97
12–18	1805	57.72
Place of residence		
Village	1603	51.26
City under 20,000 residents	414	13.24
City with 20,000 to 100,000 residents	793	25.36
City over 100,000 residents	317	10.14
Medical history		
Children with chronic disease	638	20.40
Children without chronic disease	2489	79.60

**Table 2:** Screen time in relation to the age of children.

Age group	Screen Time on Educational Activities During a Weekday		
	1–2 h n (%)	2–4 h n (%)	>4 h n (%)
<3 years old	0 (0.00%)	0 (0.00%)	1 (0.05%)
3–6 years old	11 (5.16%)	3 (1.36%)	0 (0.00%)
7–11 years old	190 (89.20%)	149 (67.73%)	520 (23.47%)
12–18 years old	12 (5.63%)	68 (30.91%)	1695 (76.49%)
**Statistical analysis**	Chi^2^ = 644.83; *p* < 0.001		
Age group	**Screen time on educational activities during the weekend day**		
	1–2 h n (%)	2–4 h n (%)	>4 h n (%)
<3 years old	1 (0.08%)	0 (0.00%)	0 (0.00%)
3–6 years old	4 (0.31%)	3 (0.40%)	0 (0.00%)
7–11 years old	494 (37.68%)	152 (20.43%)	35 (10.77%)
12–18 years old	812 (61.94%)	589 (79.17%)	290 (89.23%)
Statistical analysis	Chi^2^ = 130.67; *p* < 0.001		
Age group	**Screen time on recreational activities during the weekday**		
	1–2 h n (%)	2–4 h n (%)	>4 h n (%)
<3 years old	10 (0.74%)	2 (0.21%)	1 (0.15%)
3–6 years old	88 (6.48%)	25 (2.58%)	2 (0.31%)
7–11 years old	627 (46.17%)	338 (34.92%)	115 (17.77%)
12–18 years old	633 (46.61%)	603 (62.29%)	529 (81.76%)
Statistical analysis	Chi^2^ = 245.74; *p* < 0.001		
Age group	**Screen time on recreational activities during the weekend day**		
	1–2 h n (%)	2–4 h n (%)	>4 h n (%)
<3 years old	10 (0.88%)	2 (0.19%)	1 (0.13%)
3–6 years old	56 (4.95%)	64 (6.00%)	4 (0.53%)
7–11 years old	506 (44.74%)	416 (39.02%)	168 (22.28%)
12–18 years old	559 (49.43%)	584 (54.78%)	581 (77.06%)
Statistical analysis	Chi^2^ = 168.79; *p* < 0.001		

**Table 3:** Association between the exposure to screen during meals and screen time.

Exposure to Screens During Meals	Screen Time on Educational Activities During a Weekday		
	1–2 h n (%)	2–4 h n (%)	>4 h n (%)
**Every day**	14 (6.57%)	17 (7.73%)	273 (12.31%)
Often (several times a week)	38 (17.84%)	33 (15.00%)	616 (27.79%)
Sometimes (several times a month)	70 (32.86%)	77 (35.00%)	734 (33.11%)
Rarely (less than once a month)	52 (24.41%)	59 (26.82%)	396 (17.86%)
Never	39 (18.31%)	34 (15.45%)	198 (8.93%)
Statistical analysis	Chi^2^ = 62.09; *p* < 0.001		
Exposure to screens during meals	**Screen time on educational activities during a weekend day**		
	1–2 h n (%)	2–4 h n (%)	>4 h n (%)
Every day	136 (10.37%)	61 (8.20%)	81 (24.92%)
Often (several times a week)	307 (23.40%)	217 (29.17%)	99 (30.46%)
Sometimes (several times a month)	444 (33.84%)	282 (37.90%)	79 (24.31%)
Rarely (less than once a month)	284 (21.42%)	124 (16.67%)	47 (14.46%)
Never	144 (10.98%)	60 (8.06%)	19 (5.85%)
Statistical analysis	Chi^2^ = 98.51; *p* < 0.001		
Exposure to screens during meals	**Screen time on recreational activities during a weekday**		
	1–2 h n (%)	2–4 h n (%)	>4 h n (%)
Every day	79 (5.82%)	95 (9.80%)	156 (24.11%)
Often (several times a week)	239 (17.60%)	291 (30.03%)	235 (36.32%)
Sometimes (several times a month)	499 (36.75%)	360 (37.15%)	147 (22.72%)
Rarely (less than once a month)	339 (24.96%)	151 (15.58%)	78 (12.06%)
Never	202 (14.87%)	72 (7.43%)	31 (4.79%)
Statistical analysis	Chi^2^ = 341.02; *p* < 0.001		
Exposure to screens during meals	**Screen time on recreational activities during a weekend day**		
	1–2 h n (%)	2–4 h n (%)	>4 h n (%)
Every day	67 (5.92%)	75 (7.03%)	184 (24.40%)
Often (several times a week)	191 (16.89%)	283 (26.52%)	276 (36.60%)
Sometimes (several times a month)	377 (33.33%)	433 (40.58%)	193 (25.60%)
Rarely (less than once a month)	315 (27.85%)	182 (17.06%)	76 (10.08%)
Never	181 (16.00%)	94 (8.81%)	25 (3.32%)
Statistical analysis	Chi^2^ = 416.79; *p* < 0.001		

**Table 4:** Types of snacks eaten while using electronic devices.

Type of Snacks	Number of Responses * (n)	The Percentage Contribution of Snacks (%)
Fruit	2170	69.40
Cookies	1247	39.88
Crisps	1100	35.18
Salty sticks	907	29.01
Vegetables	887	28.37
Chocolate	764	24.43
Bars	563	18.00
Delicacies	437	13.98
Salty peanuts	312	9.98
Sandwiches	188	6.01
Cereals with Milk	52	1.66
Popcorn	51	1.63
Yogurts	27	0.86
Other snacks	187	5.98

* One respondent could have given more than one answer.

**Table 5:** Types of beverages while using electronic devices.

Type of Beverage	Number of Responses * (n)	The Percentage Contribution of Beverages (%)
Water	2480	79.31
Tea	1666	53.28
Fruit juices	1499	47.94
Carbonated drinks	558	18.80
Coffee	206	6.59
Energy drinks	109	3.49
Milk	47	1.50
Cocoa	60	1.92
Other drinks	133	4.25

* One respondent could have given more than one answer.

**Table 6:** Association between daily physical activity and screen time.

Daily Time on Physical Activity	Screen Time on Educational Activities During a Weekday		
	1–2 h n (%)	2–4 h n (%)	>4 h n (%)
<1 h	5 (2.43%)	18 (8.78%)	331 (15.64%)
1–2 h	135 (65.53%)	124 (60.49%)	1234 (58.32%)
2–4 h	35 (16.99%)	36 (17.56%)	353 (16.68%)
4–6 h	22 (10.68%)	18 (8.78%)	132 (6.24%)
>6 h	9 (4.37%)	9 (4.39%)	66 (3.12%)
Statistical analysis	Chi^2^ = 37.94; *p* < 0.001		
Daily time on physical activity	**Screen time on educational activities during a weekend day**		
	1–2 h n (%)	2–4 h n (%)	>4 h n (%)
<1 h	159 (12.67%)	103 (14.63%)	61 (19.87%)
1–2 h	753 (60.00%)	409 (58.10%)	177 (57.65%)
2–4 h	221 (17.61%)	128 (18.18%)	36 (11.73%)
4–6 h	76 (6.06%)	47 (6.68%)	20 (6.51%)
>6 h	46 (3.67%)	17 (2.41%)	13 (4.23%)
Statistical analysis	Chi^2^ = 18.57; *p* = 0.02		
Daily time on physical activity	**Screen time on recreational activities during a weekday**		
	1–2 h n (%)	2–4 h n (%)	>4 h n (%)
<1 h	120 (9.40%)	107 (11.59%)	136 (21.97%)
1–2 h	793 (62.15%)	555 (60.13%)	337 (54.44%)
2–4 h	232 (18.18%)	170 (18.42%)	83 (13.41%)
4–6 h	84 (6.58%)	72 (7.80%)	40 (6.46%)
>6 h	47 (3.68%)	19 (2.06%)	23 (3.72%)
Statistical analysis	Chi^2^ = 77.01; *p* < 0.001		
Daily time on physical activity	**Screen time on recreational activities during a weekend day**		
	1–2 h n (%)	2–4 h n (%)	>4 h n (%)
<1 h	102 (9.44%)	98 (9.81%)	159 (22.08%)
1–2 h	652 (60.31%)	628 (62.86%)	397 (55.14%)
2–4 h	208 (19.24%)	175 (17.52%)	101 (14.03%)
4–6 h	82 (7.59%)	68 (6.81%)	44 (6.11%)
>6 h	37 (3.42%)	30 (3.00%)	19 (2.64%)
Statistical analysis	Chi^2^ = 78.25; *p* < 0.001		

## Data Availability

The datasets analyzed during the current study are available from the corresponding author on reasonable request.
